# Empirical charges for chemoinformatics applications

**DOI:** 10.1186/1758-2946-6-S1-P60

**Published:** 2014-03-11

**Authors:** Tomáš Bouchal, Radka Svobodová Vařeková, Tomáš Raček, Crina-Maria Ionescu, Stanislav Geidl, Aleš Křenek, Jaroslav Koča

**Affiliations:** 1 National Centre for Biomolecular Research, Faculty of Science and CEITEC - Central European Institute of Technology, Masaryk University, Brno, 625 00, CZ, Czech Republic; 2 Institute of Computer Science and Faculty of Informatics, Masaryk University, Brno, 602 00, CZ, Czech Republic

## 

Partial atomic charges describe the distribution of electron density in a molecule, and therefore they provide clues regarding the chemical behaviour of molecules. Atomic charges are frequently used in molecular modelling applications such as molecular dynamics, docking, conformational searches, binding site prediction, etc. Recently, partial atomic charges have also become popular chemoinformatics descriptors [1].

Partial atomic charges cannot be determined experimentally, and they are also not quantum mechanical observables. For this reason, many different methods have been developed for their calculation. These charge calculation methods can be divided into two main groups, namely quantum mechanical (QM) approaches and empirical approaches. QM approaches provide accurate charges, but they are very slow and therefore not feasible for large sets of molecules. Empirical charges can be calculated quickly and their accuracy is similar to QM, making empirical charges more appropriate for chemoinformatics applications. A very useful empirical charge calculation method is EEM (Electronegativity Equalization Method) [2,3]. This method provides charges comparable to the QM approach for which the given EEM model was parameterized. The weak point of this empirical method, as well as of other empirical methods, is the necessity for parameterization, and also the insufficient coverage of currently available EEM model parameters.

In our work, we first analysed, how applicable are currently published EEM parameters in chemoinformatics. Specifically, how many molecules from databases of known organic compounds (Pubchem, ZINC, Drugbank etc.) they can cover. We found, the coverage is about 50-75%. We would like to show a methodology for preparation of parameters with higher coverage (>95% of molecules) and also its results.

## References

[B1] Todeschini R Consonni V Molecular descriptors for chemoinformatics 2009 Wiley

[B2] Mortier WJ Vangenechten K Gasteiger J Electronegativity Equalization - Application and Parametrization J Am Chem Soc 1985 107 829 835 10.1021/ja00290a017

[B3] Svobodova Varekova R Jirouskova Z Vanek J Suchomel S Koca J Electronegativity equalization method: Parameterization and validation for large sets of organic, organohalogene and organometal molecule Int J Mol Sci 2007 8 572 582 10.3390/i8070572

